# Microdroplet PCR in Microfluidic Chip Based on Constant Pressure Regulation

**DOI:** 10.3390/mi14061257

**Published:** 2023-06-15

**Authors:** Luyang Duanmu, Yuanhua Yu, Xiangkai Meng

**Affiliations:** 1 School of Physics, Changchun University of Science and Technology, Changchun 130022, China; 2 School of Life Science and Technology, Changchun University of Science and Technology, Changchun 130022, China; mxk2018@cust.edu.cn

**Keywords:** constant pressure regulation, microdroplet digital PCR, fluorescence detection, quantitative detection

## Abstract

A device and method for the constant pressure regulation of microdroplet PCR in microfluidic chips are developed to optimize for the microdroplet movement, fragmentation, and bubble generation in microfluidic chips. In the developed device, an air source device is adopted to regulate the pressure in the chip, such that microdroplet generation and PCR amplification without bubbles can be achieved. In 3 min, the sample in 20 μL will be distributed into nearly 50,000 water-in-oil droplets exhibiting a diameter of about 87 μm, and the microdroplet will be subjected to a close arrangement in the chip without air bubbles. The device and chip are adopted to quantitatively detect human genes. As indicated by the experimental results, a good linear relationship exists between the detection signal and DNA concentration ranging from 10^1^ to 10^5^ copies/μL (R^2^ = 0.999). The microdroplet PCR devices based on constant pressure regulation chips exhibit a wide variety of advantages (e.g., achieving high pollution resistance, microdroplet fragmentation and integration avoidance, reducing human interference, and standardizing results). Thus, microdroplet PCR devices based on constant pressure regulation chips have promising applications for nucleic acid quantification.

## 1. Introduction

Polymerase chain reaction (PCR), highlighted by widespread application in genetic analysis and disease diagnosis, has aroused wide attention and research interest over the past few years [1,2,3]. Based on the digital PCR developed from microfluidic technology, the latest generation of PCR technology is of smaller reaction volume, faster reaction speed, lower system noise, and higher sensitivity than conventional qPCR. Digital PCR currently falls into two types, i.e., chamber-based dPCR (cdPCR) and droplet-based dPCR (ddPCR). To be specific, cdPCR allocates nucleic acids into discrete micropores or microchambers, and ddPCR distributes the target into considerable independent uniform picoliter or nanoliter droplets [4]. In general, ddPCR is capable of achieving higher reaction volumes at lower costs than the former. Digital polymerase chain reaction (PCR) amplifies separated targets in numerous microreactors and applies Poisson statistics to quantify DNA concentration. Kinzler and Vogelstein et al. reported the principle of digital PCR for the first time [2]. Featuring superior characteristics (e.g., absolute quantification of nucleic acid, higher reproducibility than real-time PCR, and lower susceptibility to inhibition), the strength tool has been extensively employed to investigate circulating tumor DNA [5], copy number variation [6], gene expression [7], rare mutation detection [8], single cell analysis [9,10], and trace nucleic acid detection [11].

It is worth noting that stabilizing considerable monodisperse droplets is a significant challenge for ddPCR applications. Droplets must withstand critical conditions throughout the entire PCR process, including rapid temperature cycling and complex aqueous samples [12]. Accordingly, several key issues limit the further application of ddPCR, including bubble generation and evaporation. To solve the problem of evaporation, oil seals can be used [13]. However, dealing with the generation of bubbles is still being actively explored. The formation of bubbles is a common cause of microfluidic issues and one of the main reasons for PCR failure on chips [14].

There are existing bubbles and newly generated bubbles in the ddPCR process, including bubbles mixed from the sample [15] and residual microbubbles in the gaps of the chip microstructure [16]. The newly generated bubbles are mainly generated during the rapid heating and cooling process of PCR, due to the decreased gas solubility of oil at high temperatures, resulting in the formation of bubbles during oil degassing [17]. In the heating process, the bubbles mixed into the droplets are likely to move, gather, and grow; move the solution out of the reaction chamber; or apply a shear force on the droplets, thus triggering droplet integration and problems in the reading process, such that the performance and outcome of the entire PCR can be determined [17]. Indeed, the limitations of bubbles have been recognized by society, and some studies have been conducted on bubble removal. The bubble removal mechanism in microsystems conforms to membranes, porous structures, capillary interactions and/or diffusion, and the application of vacuum in the past, as reviewed by Xu et al. [18,19]. The above-mentioned methods ensure the PCR process by eliminating bubbles, whereas there are numerous challenges (e.g., the complex and cumbersome processes failed to industrialize on a large scale and to apply subsequent fluorescence detection).

To address the problems mentioned above, this study proposes a novel constant pressure control device that employs positive pressure injection to generate microdroplets at the microdroplet generation stage. Moreover, the method of constant pressure regulation of microdroplets in microfluidic chips is adopted during the PCR process to suppress the precipitation and growth of bubbles, such that the stability of microdroplets can be enhanced. The device achieves microdroplet generation, PCR amplification, and chip fluorescence signal reading on the same chip, avoiding the effect of bubbles in the experimental process. In addition, the performance of the chip-based constant pressure regulatory device in nucleic acid quantitative detection is verified by detecting the epidermal growth factor receptor (EGFR) gene, which can serve as a molecular indicator for the early detection of mutated DNA. The detection of DNA mutations can serve as a molecular indicator for the early detection of tumors and can also guide the use of molecular targeted therapies [20]. Thus, the study of droplet PCR devices in constant pressure-regulated microfluidic chips provides guidance for the in-depth practical application of droplet digital PCR chips.

## 2. Materials and Methods

### 2.1. Reagents and Instruments

The reagents and instruments used were as follows: droplet generation oil for probes (1863005, BIO-RAD, Hercules, CA, USA), forward primers (YUANQI-BIO, Shanghai, China), reverse primers (YUANQI-BIO), MGB probes (YUANQI-BIO), pGEM plasmids embedded in EGFR exon gene 18 sequence (YUANQI-BIO), ddPCRSupermix for probes (BIO-RAD); ddPCR gene chip reader (Changchun Jite, Changchun, China), Manta g-1236 CMOS camera, 200 μL centrifuge tube (AXYGEN, Hangzhou, China), micropipette (Eppendorf, Hamburg-Nord, Germany), suction head (AXYGEN), handheld centrifuge (Eppendorf), pressure calibrator (DPI611-07G), temperature calibrator (FLUKE, Everett, WA, USA), Peltier (ETX6-12-F1-3030-TA-RT-W6), flexible trachea (SMC), aluminum plate, PT1000, TM115 temperature control module (Chengdu Yexian, Chengdu, China), 42 stepper motor, sealed with rubber hole plug, ultraclean workbench (SW-CJ-1F), silicon wafer, photoresist SU-8 (Micro Chem 2050, Adel, GA, USA), cyclic olefin copolymer (TOPAS 5031), (tridecafluoro-1,1,2,2,2-tetrahydrooctyl) trichlorosilane (United Chemical Technologies, Inc., Bristol, PA, USA), electrofluorescence microscope (BX63, Olympus Corp., Tokyo, Japan) used to observe and image the experiments, dryer (SUPO), UV lamp, desktop homogenizer (MX-RLE), incubator (HZP-250), plasma cleaning machine (PTL-VM500), proportional valve (Parker), pressure sensor (MPX5050GP), and solenoid valve (Parker). ImageJ v4.4 software is adopted to process images captured in experiments.

### 2.2. Design and Fabrication of the Integrated ddPCR Gene Chip

**Figure 1:**
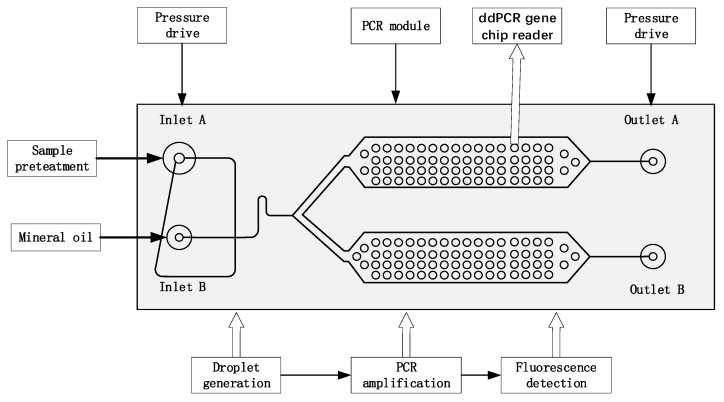
Layout of the integrated droplet digital polymerase chain reaction (ddPCR) gene chip for droplet generation, collection, on-chip thermal cycling, and fluorescence readout.

Figure 1 presents the composition and working principle of the ddPCR integrated gene chip, which achieves droplet generation, PCR amplification, and fluorescence signal reading on the same chip.

**Figure 2:**
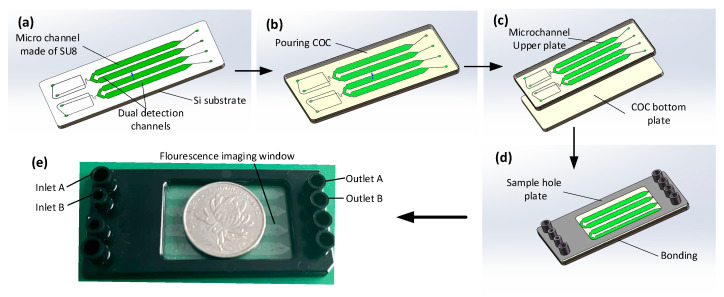
Fabrication process of the integrated droplet digital polymerase chain reaction (ddPCR) gene chip. Mold manufactured by photolithography (**a**). Injecting cyclic olefin copolymer (COC) (**b**). Bonding (**c**). Sketch of the chip (**d**). Image of the chip (**e**).

The integrated ddPCR gene chip comprises two parts, i.e., a droplet generation part and a droplet collection chamber with a height of 60 um. Solidworks 2018 software was employed to design the integrated chip structure diagrams. The microfluidic chips were manufactured using conventional photolithography methods [21,22,23,24,25] and COC injection molds. Figure 1 illustrates the design of the microfluidic chip. The main mold of the equipment was manufactured using the conventional SU8 lithography process. After the development process, the main mold was hard functionalized at 150 °C for 30 min and salted with (tridecafluoro-1,1,2,2-tetrahydrooctyl) trichlorosilane (United Chemical Technologies, Inc., Bristol, PA, USA) for 5 min to prevent pattern removal during polymer replication (Figure 2a). Subsequently, the COC particles were placed into the injection molding machine (CT80M8), the mold temperature was set to 60 °C, the melt temperature was set to 215 °C, the injection speed was set to 97 mm/s, the injection pressure was set to 60 MPa, and the pressure setting was kept at 60 mPa. The molten COC entered the mold under the push of the screw, and the chip structure layer with microchannel structure was obtained after cooling (Figure 2b). Holes with diameters of 1 mm were punched using biopsy punches. The preparation of COC chips was achieved by hot pressing and bonding the chip structure layer with another COC plastic sheet after oxygen plasma treatment (PDC-MG, Harris plasma, Union City, CA, USA) for 1 min and baking for 6 min (Figure 2c,d). The chip was placed in an oven at 105 °C for 5 h after the production was achieved to enhance the hydrophobicity of the inner wall of the microchannel and ensure the stability of the droplets. The real chip is illustrated in Figure 2e.

### 2.3. Methods

#### 2.3.1. Bubble Dynamics Equation

Bubble dynamics is mainly the study of the dynamic growth or reduction of bubbles in liquids. The Rayleigh–Plesset equation is a classic equation that describes the dynamic changes of bubbles, and its derivation assumptions are as follows:

1. The liquid is incompressible Newtonian fluid.
2. Gravity can be ignored.
3. The gas in the bubble is composed of liquid vapor and non-condensable gas. The liquid vapor in the bubble is in a saturated state, and its partial pressure is the saturated vapor pressure. The non-condensing gas content in the bubble is constant.
4. The inertia of gas and the heat exchange between gas and liquid can be ignored.

**Figure 3:**
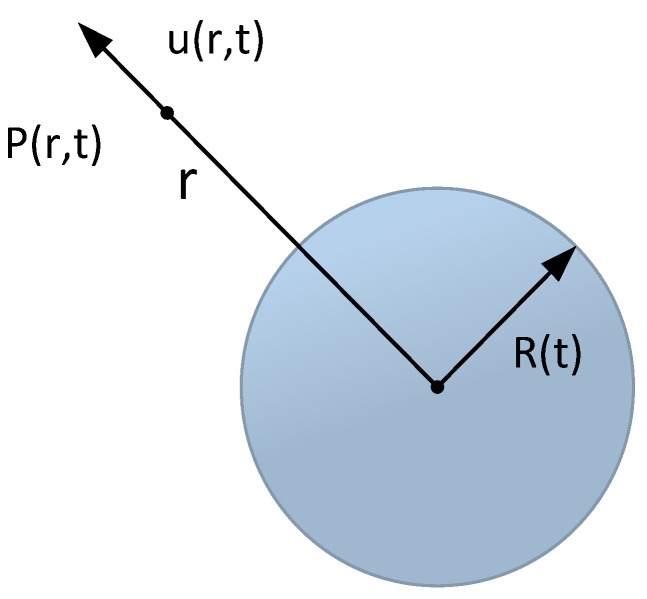
Bubble diagram.

The spherical coordinates with the center of the bubble as the origin are set, as presented in Figure 3. During the bubble change, *R*(*t*) denotes the radius of the bubble. *P*(*r*,*t*) denotes the pressure at any point r in the liquid, and *u*(*r*,*t*) expresses the velocity, such that the Rayleigh–Plesset equation can be deduced to express the dynamic behavior of bubbles as follows:

(1) $$\rho \left(R\ddot{R}+\frac{3}{2}{\dot{R}}^{2}\right)=P_{v}-P_{f}\left(t\right)+P_{g0}{\left(\frac{R_{0}}{R}\right)}^{3}-\frac{2\sigma }{R}-4\mu_{0}\frac{\dot{R}}{R}$$

#### 2.3.2. Dissolution and Precipitation of Gases in Liquids

When the Rayleigh–Plesset equation is derived, the gas content in the bubble is assumed to be constant. For practical gas–liquid two-phase fluids, gases generally exist in liquids in two forms. To be specific, Form I is suspended in the form of bubbles in the liquid; Form II dissolves into the liquid. Accordingly, the Rayleigh–Plesset equation ignores the effect of gas dissolution and precipitation on bubble dynamics. In addition, the dissolution of gas also affects the steady-state radius of bubbles when the pressure changes.

Henry’s law describes the equilibrium state of dissolution. When dissolution equilibrium occurs, the solubility of a gas is directly proportional to the pressure of the gas on the liquid surface.

**Figure 4:**
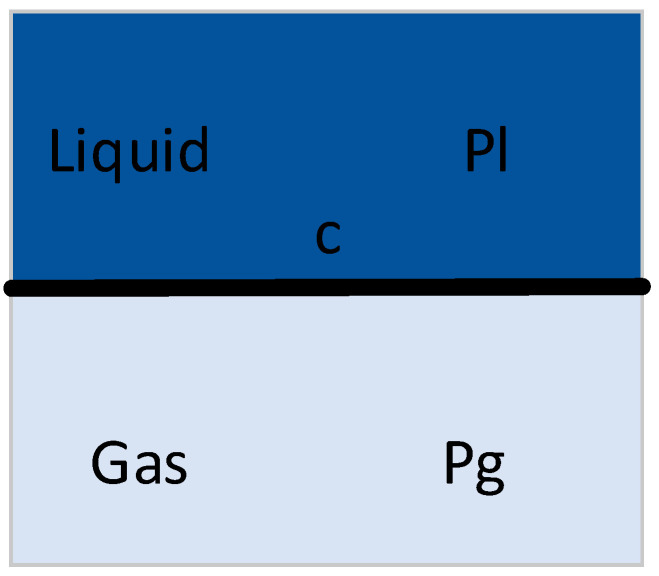
Schematic diagram of diffusion equilibrium at gas–liquid interface.

As depicted in Figure 4, the gas pressure $P_{g}$ is directly proportional to the solubility c of gas in the liquid, where

(2) $$c=\frac{P_{g}}{H}$$

where H is the Henry constant.

When calculating the effect of gas dissolution on the evolution of bubble radius, it is necessary to clarify the diffusion law of gas between bubbles and liquids. Fick’s diffusion law is a macroscopic law that describes the phenomenon of material diffusion, representing the direct ratio between the flow rate of diffusing substances per unit cross-sectional area perpendicular to the diffusion direction per unit time and the concentration gradient of diffusing substances at the interface. Fick’s diffusion law can be expressed as follows:

(3) $$J_{D}=-D_{g}\frac{\partial c}{\partial r}$$

where $J_{D}$ denotes the diffusion flow density, with the unit of $\mathrm{Kg}/m^{2}\cdot s$; $D_{g}$ represents the diffusion coefficient, with the unit of $m^{2}/s$; and *c* expresses the concentration of gas in the liquid, with the unit of $\mathrm{Kg}/m^{3}$.

The diffusion coefficient in Equation (3) is an approximate function of temperature and pressure in the bubble.

(4) $$D_{g}=\frac{2.256}{P_{g}}{\left(\frac{T}{256}\right)}^{1.81}$$

#### 2.3.3. Bubble Dynamics Equation Based on Diffusion

There is a limited effect of steam in the bubble on the dynamic process of bubbles containing non-condensable gases. Thus, assuming that the bubble is filled with non-condensing gas and does not contain liquid vapor, where

(5) $$P_{v}=0$$

there will be no continuously dissolved or precipitated gases in the practical solution. Accordingly, it is assumed that there is a limited area around the bubble that is capable of affecting gas diffusion or dissolution, where

(6) $$\rho \left(R\ddot{R}+\frac{3}{2}{\dot{R}}^{2}\right)=P_{g}-P_{f}-\frac{2\sigma }{R}-4\mu_{0}\frac{\dot{R}}{R}$$

(7) $$\frac{dP_{g}}{dt}=\frac{3}{R}\left[-R^{*}T\frac{2.256\left(\frac{P_{g}}{H_{m}}-c_{r}\right)}{M_{g}P_{g}\Delta r}{\left(\frac{T}{156}\right)}^{1.81}-P_{g}\dot{R}\right]$$

(8) $$\frac{dP_{g}}{dt}=\frac{3}{R}\left[-R^{*}T\frac{2.256\left(\frac{P_{g}}{H_{m}}-c_{r}\right)}{M_{g}P_{g}\Delta r}{\left(\frac{T}{156}\right)}^{1.81}-P_{g}\dot{R}\right]$$

### 2.4. Constant Pressure Control Device

**Figure 5:**
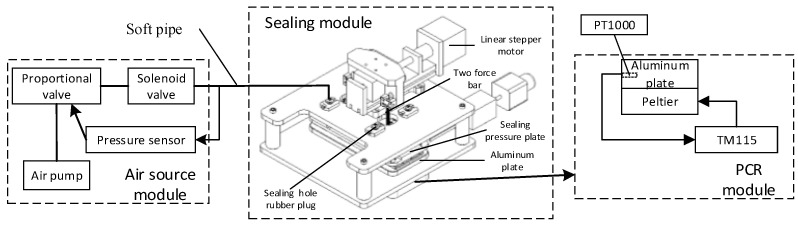
Schematic diagram of constant pressure control device.

Figure 5 presents a schematic diagram of the experimental device, which comprises the PCR module, the sealing module, and the gas source module.

The PCR module provides the rapid temperature variation for nucleic acid sample amplification, which primarily comprises a heating module and a temperature control module. The heating module covers two Parcel patches placed side by side on the aluminum plate, and the two Parcel patches are connected to the power supply in series. Moreover, the temperature control module employs the TM115 module of Chengdu Yexian Technology. In the above module, PT1000 is adopted to collect the temperature of aluminum plates. On the basis of proportional–integral (PID) closed-loop control, this module is capable of generating rapid rise and fall and uniform temperature, such that the surface temperature uniformity of aluminum blocks is achieved within ±0.3 °C.

The sealing module drives the sealing pressure plate for sealing the microfluidic chip, primarily comprising a sealing pressure plate, a two-force rod, a linear stepper motor, and four sealing rubber hole plugs. The sealing rubber hole plug is connected to the air source module through a flexible gas pipe, and driven by a linear actuator, the sealing pressure plate is pressed down. Thus, the sealing rubber hole plug is tightly attached to the inlet and outlet of the chip, ensuring the airtightness of the gas path during droplet generation and PCR.

The air source module provides stable pressure for droplet generation and PCR (e.g., an air pump, MFS flow sensor, a proportional valve, and a solenoid valve). The module can generate stable air pressure through proportional–integral (PID) closed-loop control, and the fluctuation range of droplet formation only reaches ±0.1 millibar. The chip fixture is adopted to fix the droplet generation chip, and it is capable of adjusting the air pressure through a potentiometer. When the instrument operates continuously for 2 h, the fluctuation falls into a scope of ±1 millibar.

### 2.5. Data Acquisition and Analysis

After amplification, the PCR transfers the chip to a gene chip image reader for observation. Stimulated by 485 nm (FAM) excitation, the fluorescence microscope imaging system and CMOS image sensor are employed to take photos for capturing the fluorescence images of PCR reaction results. Moreover, ImageJ software is applied to count the number of positive droplets and total droplets [26]. According to Poisson’s statistical principle, the absolute amount of target DNA contained in the respective reaction can be calculated using Equation (1) and the practical number of positive droplets [27]:

(9) c=−[n×ln(1−dn)]Vd×n

where c is the concentration of target DNA in the sample, n is the total number of droplets in the chip, d is the number of positive droplets, $V_{d}$ is the volume of each droplet.

## 3. Results and Discussion

### 3.1. Microdroplet Generation and Collection

**Figure 6:**
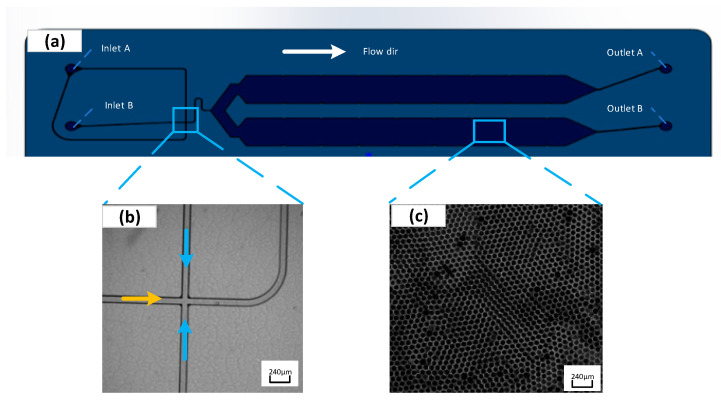
Schematic diagram of the integrated droplet digital polymerase chain reaction (ddPCR) gene chip structure (**a**). Flow-focusing structure of droplet generation part (blue arrows represent the flow direction of the oil; orange arrow represents the flow direction of the sample) (**b**). Single-layer arrangement of droplets in the collection chamber (**c**) (color figure online).

Figure 6b presents the flow-focusing structure generated by liquid droplets. The oil phase wraps the sample into droplets at the intersection of the flow-focusing structure and under the combined action of surface tension and shear force. The chip adopts a positive pressure injection method, and the droplet diameter is primarily determined by the size of the flow-focusing structure and the flow rate ratio of the discrete phase (sample) to the continuous phase (oil). Furthermore, the droplets generated in the collection chamber are arranged in a single layer.

**Figure 7:**
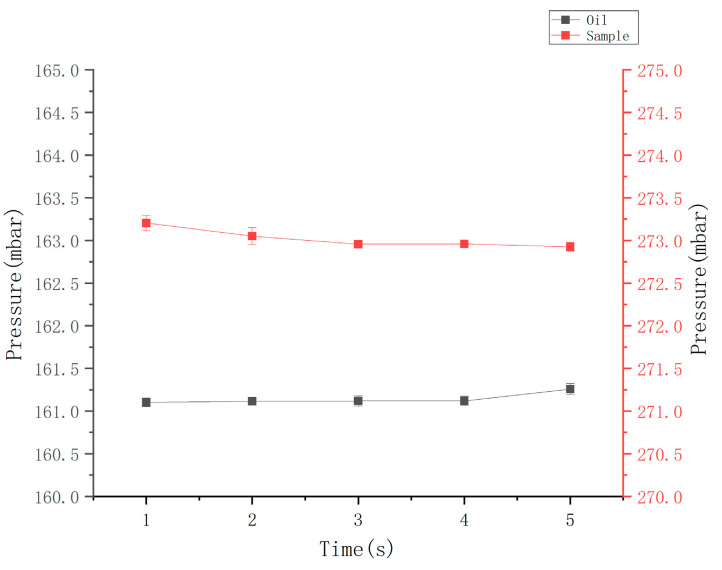
Pressure map during microdroplet generation.

In this study, the expected diameter of the microdroplets was 80 ± 10 μm, and the microchannel size was determined to be square in cross-section and 80 μm × 80 μm in size. The height of the collection chamber was smaller than the droplet diameter to avoid the appearance of overlapping droplets in images. Prior to the droplet generation, the generated oil was applied to wet the entire flow channel, which is conducive to reducing bubbles in the chip flow channel. Subsequently, 20 μL of sample and 70 μL of oil were introduced to the chip sampling hole, and the pressures at the oil inlet and sample inlet were set to 161 mbar and 273 mbar, respectively. The pressure calibrator was adopted to verify the set air pressure of the device, and the results are presented in Figure 7, suggesting that the pressure falls into a scope of the set ±0.3 mbar.

In the experiment, the sample was dispersed into droplets of approximately 50,000 droplets within 3 min while entering the droplet collection chamber, following the microchannel under positive pressure and capillary action (Figure 6c). ImageJ software was adopted to process images captured from experiments. The diameter of the generated droplets can be obtained through experiments after several settings (e.g., the binarization, edge searching, and threshold adjustment). As revealed by the experimental results, the average diameter of the droplets is approximately 87 μm. The standard deviation of the average diameter reaches 1.02 μm, the coefficient of variation (CV%) is nearly 0.91%, and the volume of each droplet is about 0.27 nL, confirming that the droplet generation exhibits excellent uniformity and competence in conforming to the needs of biochemical experimental detection and analysis.

### 3.2. Bubble Suppression during PCR Process

As revealed by the analysis in accordance with the bubble behavior theory in Section 2.3, during the whole PCR process, with the cyclic change in temperature from 45 °C to 95 °C, the amount of dissolved gas in the solution is decreased, and the solution is degassed to form bubbles, such that microdroplets are destroyed. Degassing is the main reason for forming bubbles. The force analysis of bubbles in the solution shows that there is a pressure value that makes the solution not degassed with the elevation of the temperature, and no bubbles are produced in the PCR process.

**Figure 8:**
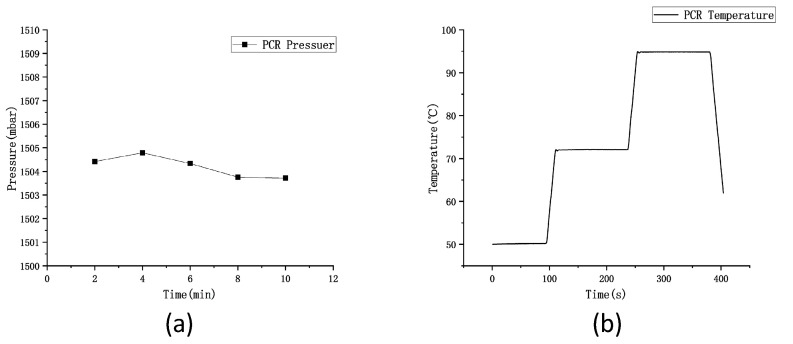
Pressure and temperature map for PCR. (**a**) Air pressure of the device during PCR. (**b**) Temperature of aluminum plate surface during PCR.

Different pressure values (0 mbar, 700 mbar, 1500 mbar, and 2000 mbar) were set in this study in accordance with the theory, and the device’s air pressure was calibrated using a pressure calibrator. As indicated by the results, the average pressure is 1504.2 mbar in 15 min and fluctuates within ±0.2 mbar in 10 min, as presented in Figure 8a. Next, the generated chip was placed in the device, and the PCR process and 40 temperature cycles were set. The Peltier power reached 120 W, which could achieve an average temperature rise and fall rate of 2.5 °C/s on the surface of the aluminum plate. In the PCR process, 50 °C, 72 °C, and 95 °C were set, and the temperature on the surface of the aluminum plate over time is presented in Figure 8b. As indicated by the results, the deviation between the surface temperature of the aluminum plate and the set temperature was within 0.5 °C, and the temperature uniformity of six random points on the aluminum plate surface was examined within a scope of 0.5 °C.

**Figure 9:**
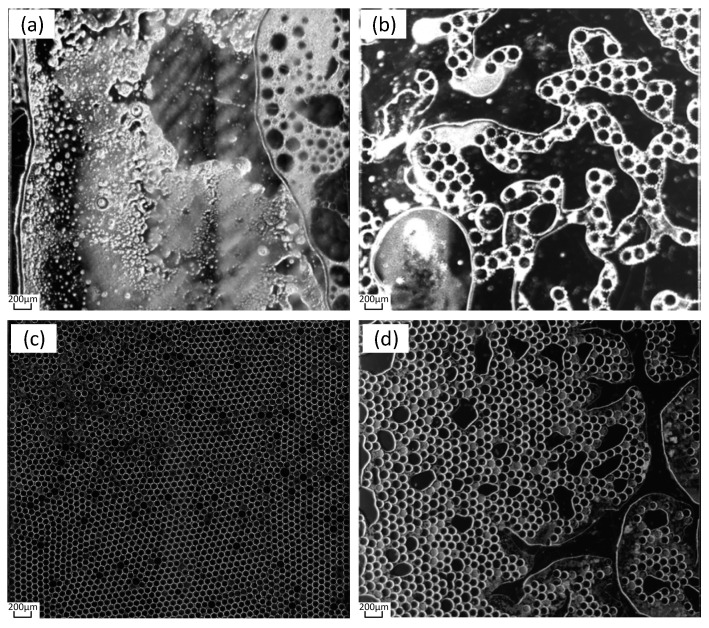
PCR Results under Different Pressures. (**a**) PCR results at 0 mbar. (**b**) PCR results at 700 mbar. (**c**) PCR results at 1500 mbar. (**d**) PCR results at 2000 mbar.

During the PCR, the pressure slowly increased to 1500 mbar as the temperature increased. After reaching 1500 mbar, the pressure continued until the end of the PCR process. After completing the PCR process, we continued to maintain it for 5 min, and then we slowly reduced the pressure to atmospheric pressure. The experimental results obtained after ending the pressure continuing are shown in Figure 9. The results show that most of the micro-droplets inside the chip were destroyed at the pressure of 0 mbar, and the solution dried up, as shown in Figure 9a. When the pressure was 700 mbar, large bubbles formed inside the chip, as shown in Figure 9b; when the pressure was 1500 mbar, the microdroplets inside the chip were intact, as shown in Figure 9c; when the pressure was 2000 mbar, microdroplets broke up inside the chip, as shown in Figure 9d. Although microdroplet damage occurred at both 700 mbar and 2000 mbar pressures, the reasons for the two occurrences are different. Under the pressure of 700 mbar, the formation of larger bubbles is caused by the re-precipitation of gas in the solution; at the pressure of 2000 mbar, excessive pressure was applied to the cavity, resulting in shear force and damage to the droplets, where no new gas was released and no large bubbles were formed.

### 3.3. Quantitative Testing

**Figure 10:**
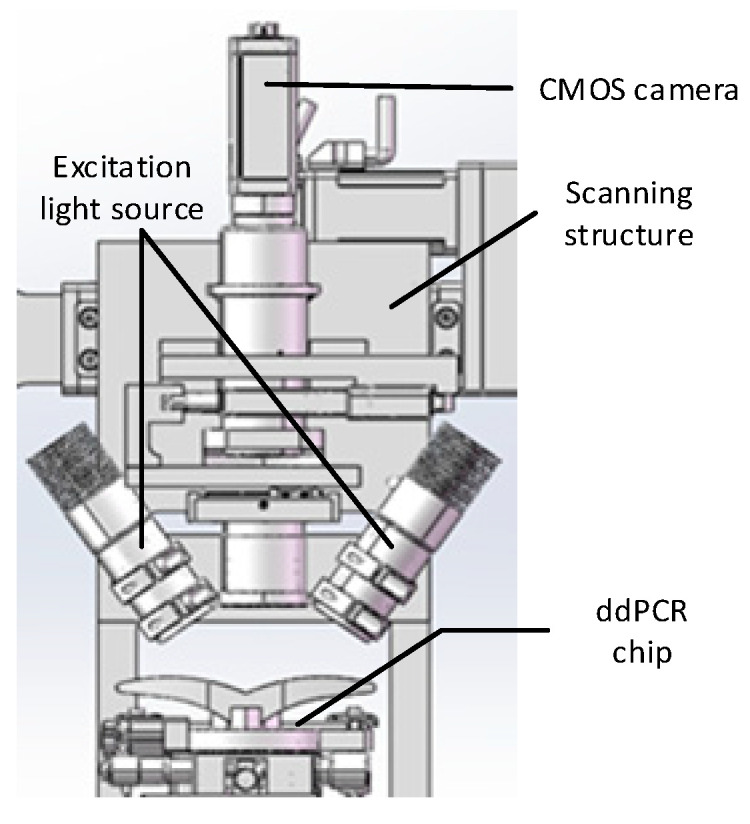
Fluorescence signal acquisition system.

**Figure 11:**
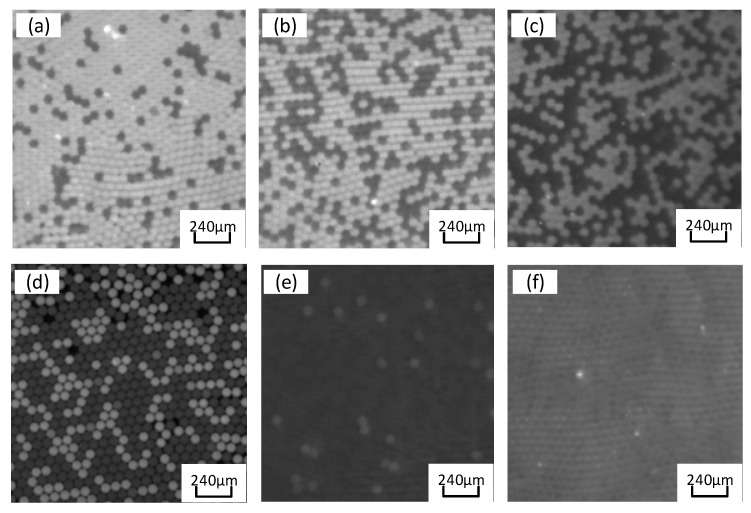
Fluorescence images for simultaneous detection of epidermal growth factor receptor (EGFR) exon 18 gene with various DNA concentrations. DNA concentration (copies/μL): (**a**) 10^5^; (**b**) 10^4^; (**c**) 10^3^; (**d**) 10^2^; (**e**) 10^1^; (**f**) negative control.

The PCR amplified fluorescence signal was examined by transferring the chip to the laser-induced fluorescence chip reader (Figure 10). The probe binds to the target gene while releasing a fluorescent reporter. Accordingly, droplets containing templates are capable of observing strong fluorescence signals under excitation light. In contrast, droplets without templates only exhibit weak background fluorescence signals. Thus, fluorescence intensity can be adopted to designate droplets as positive or negative, where the identification and statistics of negative/positive droplets take on critical significance in ddPCR technology. The grayscale values of droplets in fluorescence images were read using ImageJ v4.4 (National Institutes of Health, Bethesda, MD, USA) software, and fluorescence thresholds were determined based on differences in droplet fluorescence intensity. Droplets with fluorescence signals greater than the threshold are considered positive, whereas those below the threshold are considered negative. The quantitative detection of nucleic acid was validated using a constant pressure regulatory device based on the ddPCR gene chip in accordance with Poisson’s statistical principle. EGFR exon 18 gene served as the target DNA molecule, and the pGEM plasmid solution of EGFR exon 18 gene was diluted to generate concentration gradient standards of five orders of magnitude for 1 × 10^1^ to 1 × 10^5^ copies/μL. As depicted in Figure 11a, the positive droplets are generally saturated when the sample concentration is 10^5^ copies/μL. The number of positive droplets decreases with the template concentration from 10^5^ copies/μL to 10^1^ copies/μL (Figure 11b–e).

**Figure 12:**
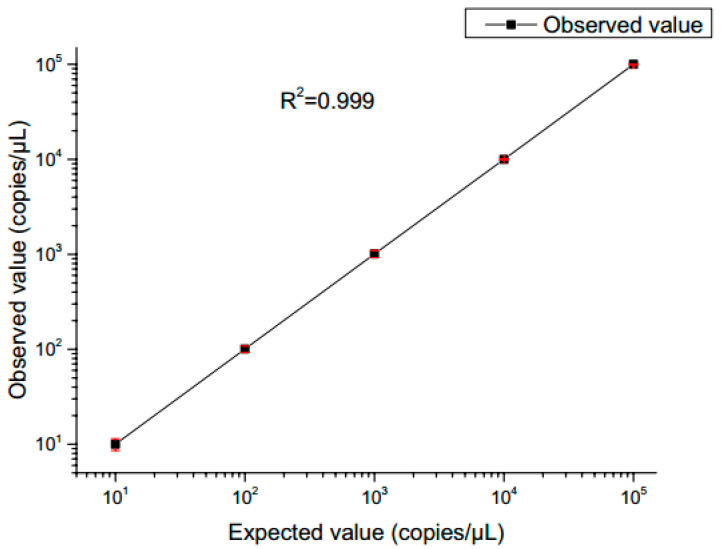
Observed value in the integrated droplet digital polymerase chain reaction (ddPCR) gene chip against the expected value. The error bars represent the 95% confidence interval.

The fluorescence images were captured after 40 thermal cycles, and the fluorescence signal was determined via the fluorescence excitation channel of EGFR exon gene 18 with light excitation at 485 nm (FAM). In accordance with Poisson’s statistical principle, Equation (9) was adopted to calculate the absolute amount of target DNA in the respective reaction system. The concentration of DNA templates was calculated based on the results of fluorescence detection systems for samples with different concentration gradients. Subsequently, the standard curve was generated (Figure 12). After the ddPCR gene chip’s performance in detecting the target gene concentration of 1 × 10^1^ to 1 × 10^5^ copies/μL was summarized, the results revealed a good linear relationship.

## 4. Conclusions

A device was developed based on a microfluidic chip with microdroplet generation and PCR constant pressure regulation; it achieves droplet generation and PCR amplification on the same chip, followed by fluorescence detection analysis. Moreover, the source of bubbles and the theory of bubble dynamic behavior in rapid temperature changes were discussed by analyzing the digital PCR process, allowing the problem of bubble growth and generation to be solved. The device is highly competent in stably generating microdroplets without bubbles in the chip while performing PCR amplification at 1500 mbar. The amplified microdroplets will not break or mix, supporting subsequent fluorescence detection. Furthermore, the device and integrated ddPCR gene chip were employed to quantitatively detect EGFR exon gene 18 fragments. As revealed by the results, a good linear correlation was generated when the DNA concentration was between 10^1^ and 10^5^ copies/μL. Accordingly, the constant pressure regulation device has promising applications in the field of nucleic acid-associated detection based on microfluidic chips.

## Data Availability

The data that support the findings of this study are available from the corresponding author upon reasonable request.
